# Gastroesophageal reflux disease symptoms and associated factors among university students in Amhara region, Ethiopia, 2021: a cross-sectional study

**DOI:** 10.1186/s12876-023-02758-8

**Published:** 2023-04-19

**Authors:** Mekonnen Belete, Winta Tesfaye, Yonas Akalu, Adugnaw Adane, Yigizie Yeshaw

**Affiliations:** 1 Department of Human Physiology, School of Medicine, College of Medicine and Health Sciences, Wollo University, Dessie, Ethiopia; 2 Department of Human Physiology, School of Medicine, College of Medicine and Health Sciences, University of Gondar, Gondar, Ethiopia; 3 Department of Epidemiology and Biostatistics, Institute of Public Health, College of Medicine and Health Sciences, University of Gondar, Gondar, Ethiopia; 4 Department of Human Physiology, School of Medicine, College of Medicine and Health Sciences, Hawassa University, Hawassa, Ethiopia

**Keywords:** GERD, GERD symptoms, University students, Amhara, Ethiopia

## Abstract

**Introduction:**

Gastroesophageal reflux disease (GERD) symptom is a relapsing chronic medical condition resulting from the reflux of gastric acid contents into the esophagus and throat or mouth. It interferes with social functioning, sleep, productivity, and quality of life. Despite this, the magnitude of GERD symptoms is not known in Ethiopia. Therefore, this study was conducted to determine the prevalence and associated factors of GERD symptoms among university students in the Amhara national regional state.

**Methods:**

An institutional-based cross-sectional study was employed in Amhara national regional state Universities, from April 1, 2021, to May 1, 2021. Eight hundred and forty-six students were included in the study. A stratified multistage sampling technique was employed. Data were collected by using a pretested self-administered questionnaire. Data were entered via Epi Data version 4.6.0.5 and analyzed by SPSS version-26 software. The bivariable and multivariable binary logistic regression analyses were used to determine the associated factors of GERD symptoms. The adjusted odds ratio (AOR) with a 95% confidence interval (CI) was calculated. Variables having a p-value of ≤ 0.05 were considered statistically significant.

**Results:**

The prevalence of GERD symptoms in this study was 32.1% (95% CI = 28.7–35.5%). Being in the age of 20–25 years (AOR = 1.74, 95%CI = 1.03–2.94), female (AOR = 1.67, 95% CI = 1.15–2.41), use of antipain (AOR = 2.47, 95% CI = 1.65–3.69) and soft drinks (AOR = 1.58, 95% CI = 1.13–2.20) were significantly associated with higher odds of GERD symptoms. Urban dwellers had less chance of having GERD symptoms (AOR = 0.67, 95% CI = 0.48–0.94).

**Conclusion:**

Nearly one-third of university students are affected by GERD symptoms. Age, sex, residence, use of antipain, and consumption of soft drinks were significantly associated with GERD. Reducing modifiable risk factors such as antipain use and soft drink consumption among students is advisable to decrease the disease burden.

## Introduction

Gastroesophageal reflux disease (GERD) is a chronic medical condition resulting from the reflux of gastric acid contents into the esophagus and throat or mouth to cause distressing symptoms /complications [1, 2]. It also develops due to sensorimotor disorder associated with impairment of the normal anti-reflux mechanisms and with changes in normal physiology [3].

The pooled global prevalence of GERD is 14% [4]. This magnitude varies by region, ranging from 2.5 to 33.1%, in North America, Europe, East Asia, the Middle East, Australia, and South America population [5]. The prevalence of GERD ranges from 11.8 to 52.6% among university students [6–13].

Gastroesophageal reflux disease is a potentially serious condition with risks of complications like stricture of the esophagus, Barrett’s esophagus (pre-cancerous lesion), and malignancy, [14] and could be turned into a life-threatening disease [15]. It had extra-esophageal complications such as chronic cough, chronic laryngitis, asthma, and dental erosions [2]. Due to its chronic pain, and persistent and disruptive symptoms, GERD can impair physical and mental health-related quality of life, workplace productivity (daily tasks), social function, sleep, and diet, as well as cause anxiety and depression [7, 10, 16, 17]. Gastroesophageal reflux disease can cause an economic burden due to the disease’s diagnostic and therapeutic management [11]. Heartburn, regurgitation of food, vomiting, and regurgitation during sleep [18] are the most common symptoms of GERD. Based on these symptoms, a clinical diagnosis of GERD could be made [19].

Sociodemographic, lifestyle, dietary, and behavioral, as well as psychological factors, are associated with GERD [4, 19–23]. These were sex, age, residence, [7, 11, 18, 19, 24–32] sleeping within 1 h of dinner [8, 20, 24].

Consumption of caffeinated and soft drinks, [8, 9, 18, 24, 33–43] and types of food consumption [8, 35, 37–39, 41–44]. Inadequate sleep, [7, 20] smoking, [8, 11, 19, 23–25, 28, 31, 36, 40, 41, 44–47] history of use of non-steroidal anti-inflammatory drugs or analgesics, [20, 21, 28, 32, 35, 45, 47–50], and alcohol consumption, [19, 20, 23, 39, 40, 46, 51] were associated with GERD symptoms.

Even though GERD symptoms can negatively impact one’s quality of life, daily tasks, and the country’s economy by requiring the purchase of medication to alleviate GERD symptoms in university students, the burden of GERD symptoms in Ethiopian university students has not been quantified. As a result, this research aimed to assess the prevalence of GERD symptoms and its associated factors among university students in Ethiopia’s Amhara area. Stakeholders will use the outcomes of this study to develop illness prevention, care, and early treatment methods.

## Methods

### Study settings, period, and design

An institutional-based cross-sectional study was employed in Amhara national regional state Universities, from April 1, 2021, to May 1, 2021. Amhara’s national regional state is in North Ethiopia and its capital city is Bahir Dar. There are 10 government-owned Universities in Amhara national regional state and, from these Universities; three of them were selected by using the lottery method for the study; namely, the University of Gondar, Wollo, and Woldia University.

### Source and study population

All government university students in the Amhara region were the source population, and all regular undergraduate students registered in the University of Gondar, Wollo, and Woldia University in the 2020/21 academic year were the study population.

### Sample size determination and sampling procedure

The sample size was calculated using single population proportion formula, with the assumption of 95% CI (Za/2 = 1.96), 5% (α = 0.05) level of significance, 5% (d = 0.05) margin of error, and proportion of 50% (no study done about GERD in Ethiopia), accordingly, the calculated sample size for the study became 384.

After adding a 10% non-response rate, the calculated sample size was 423. Since we have used multistage random sampling, this sample was multiplied by the design effect of two. Therefore, the final total sample size for this study was 846.

**Fig. 1:**
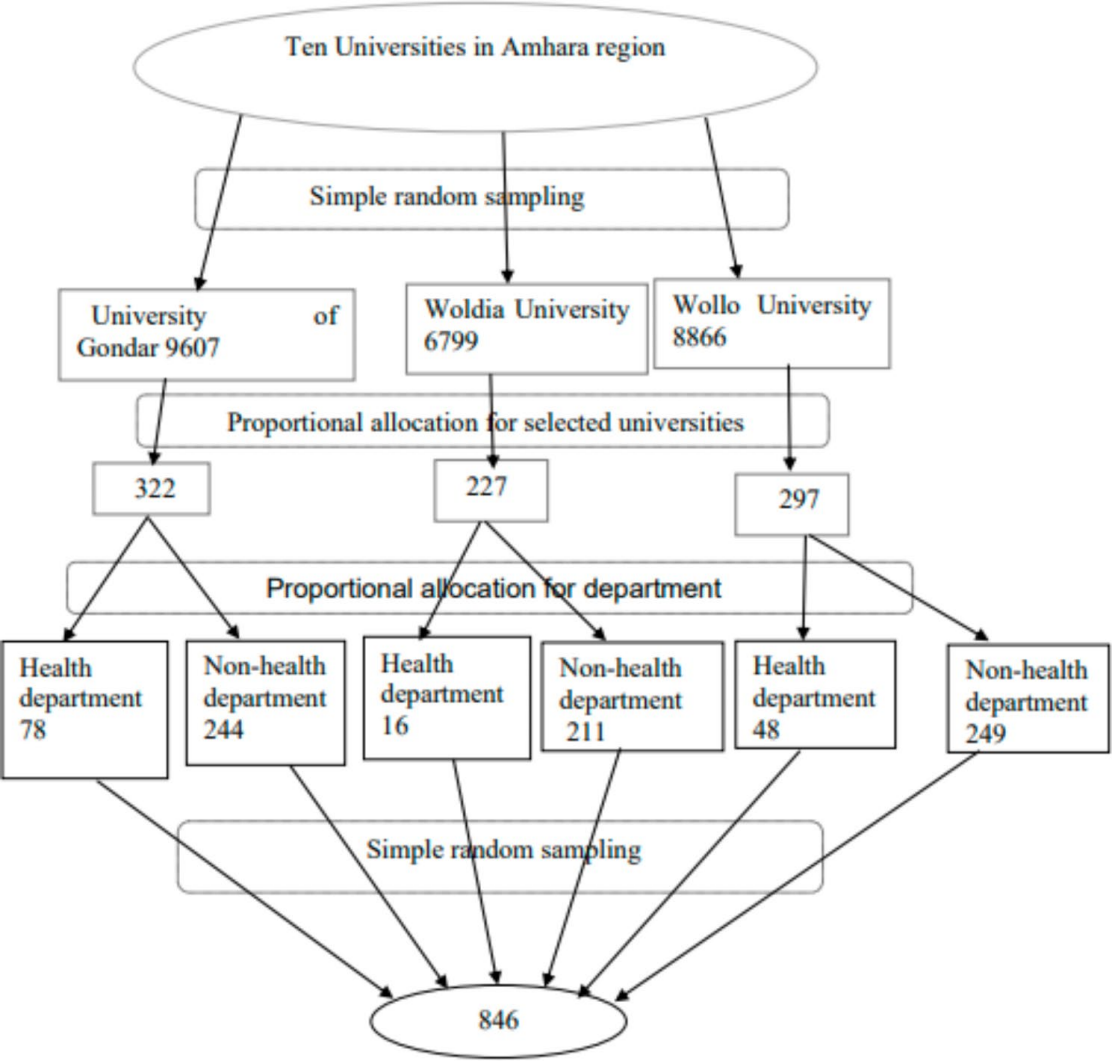
Schematic representation of sampling procedure for the prevalence and associated factors of GERD among university students in Amhara region, Ethiopia, 2021

Three Universities were chosen using a simple random selection methodology (lottery method) out of the total Universities in the Amhara region. There were 25,272 undergraduate regular program students registered at the three selected Universities. The University of Gondar had 9,607 students, whereas Wollo University had 8,866 students and Woldia University had 6,799 students. Three hundred and twenty-two students from the University of Gondar, 297 from Wollo University, and 227 from Woldia University were chosen using proportionate allocation. After that, a proportional sample of these universities was assigned to the departments (health and non-health). A simple random sample procedure was used to pick research participants from each department (Fig. 1).

### Study variables

The dependent variable was gastroesophageal reflux disease. Students were said to have GERD symptoms when the Gastroesophageal reflux disease questionnaire score was ≥ 8 [52].

The independent variables were age, sex, marital status, year of study, department, residence, types of food consumed, eating habits, skipping breakfast, the timing of sleep soft drink consumption, sleep pattern (length of sleep per day), alcohol consumption, cigarette smoking, khat chewing and history of use of non-steroidal anti-inflammatory drugs or analgesics.

#### Operational definitions

**Fasting food**-is food that is eaten during fasting and is free from dairy, egg, and meat products.

**Spicy food** -food that has been prepared with the use of spices or herbs that give it a pungent or hot flavor such as paper and chili.

**Fatty food**- any food that contains a high level of fat like dairy, egg, and meat products.

**Fried or cooked food** -is cooking of food in hot fats or oils, usually done with a shallow oil bath in a pan over a fire in which the food is completely immersed in a deeper vessel of hot oil.

#### Data collection tools and procedures

A semi-structured self-administered questionnaire and the Gastroesophageal reflux diseases questionnaire (GERDQ) were used to collect data. The GERDQ was a patient-centered, self-assessment tool that helped doctors diagnose, manage, and assess GERD symptoms without requiring an initial expert referral or endoscopy [52]. It also had diagnostic value in an unselected population presenting with typical and/or atypical GERD symptoms [53]. The participants were asked to recall their symptoms and the frequency with which they occurred throughout the previous seven days, according to GERDQ. Positive symptoms such as heartburn, regurgitation (reflux), heartburn and reflux disrupting sleep at night, and the need for further drugs were rated as follows: 0, 1, 2, and 3 points for 0 days, 1 day, 2–3 days, and 4–7 days, respectively. Second, the frequency of negative symptoms (upper abdominal pain and nausea): 3, 2, 1, and 0 points for 0 days, 1 day, 2–3 days, and 4–7 days, respectively.

The sum of the points for these frequencies served as a subject’s GERDQ scores, and GERD symptoms were diagnosed if the sum was greater than or equal to 8 points [52]. Six BSc Nurses and three BSc Public Health professionals were involved in data collection and supervision, respectively. Before collecting data, select study participants from health and non-health department based on the proportional allocation method from these selected universities. Then, select the study participants by lottery method based on their identification number which is obtained from each university’s registrar. Then after, the data collectors inform the study participants about the study’s purpose and objectives. Provide instruction to the students so they can fill out the questionnaires correctly, comprehend the questions, and avoid writing their names or other unneeded information about themselves.

### Data quality control

The questionnaire was translated from the English version into the local language (Amharic version) and then retranslated back to English to ensure consistency. A pretest was conducted on 5% of the sample at Mekdela Amba University students. Appropriate training was given to the data collectors and supervisors. The collected data were checked for completeness, consistency, and accuracy on daily basis by the principal investigator.

### Data processing and analysis

Data were entered into Epi Data version 4.6.0.5 and then, exported into SPSS version-26 software for data analysis. Bivariable binary logistic regression analysis was performed to determine the associated factors of GERD symptoms.

All variables with a p-value ≤ 0.25 at bivariable binary logistic regression analysis were entered into the multivariable binary logistic regression model. The strength of association was described by computing the odds ratio with a 95% confidence interval (CI). Variables having a p-value ≤ 0.05 in the final model were considered statistically significant.

## Results

### Sociodemographic characteristics of the respondents

Eight hundred and forty-six students were included in the study and the overall response rate was 93.5%. The age of the respondents ranges from 19 to 30 years with a median of 22 years and an interquartile range of two (2). Most of the respondents 464 (58.7%) were males and 486 (61.5%) were Orthodox Christianity followers. Most of the respondents, 705 (89.2%)) were unmarried and 648 (81.9%) were from the non-health department. Nearly half of the respondents (53.4%) were urban dwellers and 290 (36.7%) were a second year (Table 1).

**Table 1:** Socio-demographic characteristics of university students in Amhara region, Ethiopia, 2021, (n = 791)

Variables	category	Frequency	Percent (%)
Age(years)	< 20	133	16.8
	20–25	619	78.3
	> 25	39	4.9
Sex	Female	327	41.3
	Male	464	58.7
Religion	Orthodox	486	61.4
	Muslim	146	18.5
	Catholic	38	4.8
	Protestant	114	14.4
	Adventist	7	0.9
Residence	Rural	369	46.6
	Urban	422	53.4
Marital status	Married	85	10.7
	Unmarried	706	89.3
Year of Study	2	290	36.6
	3	283	35.8
	4	178	22.5
	5	40	5.1
Department	Health	143	18.1
	Non-health	648	81.9

### Lifestyle, dietary, and behavioral characteristics of the respondents

Of the total respondents, 542 (68.6%) took fasting food frequently and almost three-fourths (76.1%) of the respondents take food from university cafes. Nearly half of the respondents (52%) had the habit of eating quickly and 51.7% did not skip breakfast. Three-fourths of the respondents (74.5%) had a sleep after dinner for more than or equal to 2 h. Regarding tea and coffee consumption, 403 (51%) and 481(61%) of the respondents did not frequently drink tea and coffee, respectively. Most of the respondents (63.5%) did not take soft drinks frequently. Most of the respondents were not smokers (93.4%) and khat chewers (90.1%). Most of the respondents (62.4%) were not alcohol drinkers. Half of the respondents (50.9%) had a sleep duration of fewer than seven hours, and 646 (81.7%) students did not use antipain medication (Table 2).

**Table 2:** Lifestyle, dietary and behavioral factors of university students in Amhara region, Ethiopia, 2021, (n = 791)

Variables	Category	Frequency	Percent (%)
Types of food consumed	Fasting	542	68.5
	Spicy	96	12.1
	Fatty	73	9.2
	Fried and cooked	80	10.1
Place of feeding	University cafe	602	76.1
	Non-cafe	189	23.9
Skip of breakfast	Yes	382	48.3
	No	409	51.7
Time of sleep after dinner	< 2 h	202	25.5
	≥ 2 h	589	74.5
Quick eating	Yes	411	52
	No	380	48
Smoke cigarette	Yes	53	6.6
	No	738	93.4
Alcohol consumption	Yes	298	37.6
	No	493	62.4
Length of sleep per day	< 7 h	403	50.9
	≥ 7 h	388	49.1
Use antipain	Yes	145	18.3
	No	646	81.7
Types of antipain used	Paracetamol	93	64.1
	NSAIDs/analgesics (diclofenac, ibuprofen)	52	35.9
Chew khat	Yes	78	9.9
	No	713	90.1
Consumption of tea	Yes	387	48.9
	No	404	51.1
Consumption of coffee	Yes	309	39.1
	No	482	60.9
Soft drink consumption	Yes	289	36.5
	No	502	63.5

### Prevalence of gastroesophageal reflux diseases symptoms

The prevalence of GERD symptoms among university students in the Amhara region was 32.1%(95%CI = 28.7–35.5%). Regarding positive symptoms of GERD, 45.5% of the respondents had heartburn, 40.4% had regurgitation, 39% had sleep disturbance due to heartburn and regurgitation, and 18.2% used medication for relief from heartburn and regurgitation in the previous week. Concerning negative symptoms of GERD symptoms, 54.8% and 61% of the respondents had not experienced epigastric pain and nausea in the previous week, respectively (Table 3).

**Table 3:** Gastroesophageal reflux disease symptoms among university students in Amhara region, Ethiopia, 2021, (n = 791)

Symptoms	Category	Frequency	Percent (%)
Heartburn	None /week	431	54.5
	Once/week	120	15.1
	2–3 days/week	177	22.4
	4–7 days/week	63	8
Regurgitation	Zero-day/week	472	59.6
	One day/week	169	21.4
	2–3 days/week	117	14.8
	4–7 days/week	33	4.2
Epigastric pain	None /week	434	54.8
	One day/week	157	19.9
	2–3 days/week	142	18
	4–7 days/week	58	7.3
Nausea	None /week	483	61
	One day/week	184	23.3
	2–3 days/week	101	12.8
	4–7 days/week	23	2.9
Sleep disturbance	Zero-day/week	484	61.1
	None /week	179	22.7
	2–3 days/week	107	13.5
	4–7 days/week	21	2.7
Use medication for relief from heartburn and regurgitation	None /week	647	81.8
	One day/week	96	12.2
	2–3 days/week	36	4.6
	4–7 days/week	12	1.5

### Factors associated with gastroesophageal reflux disease

On bivariable binary logistic regression analysis, age, sex, residence, year of study, types of food consumed, place of feeding, skip breakfast, cigarette smoking, chewing khat, use of anti-pain, frequent use of coffee and tea, and soft drink were associated with GERD symptoms (p ≤ 0.25).

In multivariable binary logistic regression analysis, age, place of residence, sex, use of antipain, and soft drink were significantly associated with GERD symptoms (p ≤ 0.05). The odds of having GERD symptoms were 1.74 times higher among the age group of 20–25 years compared to those respondents aged less than 20 years (AOR = 1.74, 95% CI = 1.03–2.94). Females had a 1.67 times higher chance of having GERD symptoms than males (AOR = 1.67, 95% CI = 1.15–2.41). Students who came from urban areas had a 33% reduced chance of having GERD symptoms than rural dwellers (AOR = 0.67, 95% CI = 0.48–0.94). Students who had a history of antipain use were 2.47 times more likely to have GERD symptoms than their counterparts (AOR = 2.47, 95% CI = 1.65–3.69). Students who use soft drinks were 1.58 times more likely to have GERD symptoms than those who did not use a soft drink (AOR = 1.58, 95% CI = 1.13–2.20) (Table 4).

**Table 4:** Bivariable and multivariable binary logistic regression analysis for factors associated with Gastroesophageal reflux disease symptoms among university students in Amhara region, Ethiopia, 2021, (n = 791)

Variables	Category	GERD symptoms status		Odds Ratio (95% of CI)	
		Yes (%)	No (%)	COR	AOR
Age(years)	< 20	35 (26.3)	98 (73.7)	1.00	1.00
	20–25	208(33.6)	411 (66.4)	1.42(0.93–2.16)	1.74(1.03, 2.94) *
	> 25	11 (28.2)	28 (71.8)	1.1 (0.50–2.44)	1.32(0.53–3.31)
Sex	Female	128(39.1)	199 (60.9)	1.73(1.28–2.33)	1.67(1.15,2.41) ***
	Male	126(27.2)	338 (72.8)	1.00	1.00
Residence	Rural	126(34.1)	243 (65.9)	1.00	1.00
	Urban	128(30.3)	294 (69.7)	0.84 (0.62–1.13)	0.67(0.48,0.94) *
Type of food consumed	Fasting food	169(31.1)	373 (68.9)	1.00	1.00
	Spicy food	33 (34.4)	63(65.6)	1.12 (0.67–1.88)	0.87(0.52,1.45)
	Fatty food	29 (39.7)	44(60.3)	1.30 (0.68–2.47)	1.13(0.64,1.99)
	Fried and cooked	23 (28.7)	57(71.3)	1.63 (0.83–3.21)	0.69(0.39,1.24)
Place of food take	University cafe	184(30.6)	418(69.4)	0.75 (0.53–1.05)	1.08(0.71,1.63)
	Non-cafe	70 (37.0)	119 (63.0)	1.00	1.00
Skip breakfast frequently	Yes	132(34.6)	250 (65.4)	1.24 (0.92–1.68)	1.05(0.75,1.46)
	No	122(29.8)	287 (70.2)	1.00	1.00
Smoke cigarette	Yes	23 (43.4)	30 (56.6)	1.68 (0.96–2.96)	1.58(0.78,3.22)
	No	231(31.3)	507 (68.7)	1.00	1.00
Use pain killer	Yes	79 (54.5)	66 (45.5)	3.22 (2.23–4.66)	2.47(1.65,3.69) ***
	No	175(27.1)	471 (72.9)	1.00	1.00
Chew Chat	Yes	32 (41.0)	46 (59.0)	1.54 (0.95–2.48)	1.25(0.69,2.28)
	No	222(31.1)	491 (68.9)	1.00	1.00
consumption of tea	Yes	141(36.4)	246 (63.6)	1.48 (1.09–1.99)	1.18(0.85,1.64)
	No	113(28.0)	291(72)	1.00	1.00
consumption of coffee	Yes	117(37.9)	192 (62.1)	1.54(1.13–2.08)	1.23(0.88,1.72)
	No	137(28.4)	345 (71.6)	1.00	1.00
Consumption of soft drink	Yes	119(41.2)	170 (58.8)	1.90(1.40–2.59)	1.58(1.13,2.20) **
	No	135(26.9)	367(73.1)	1.00	1.00
Year of Study	2	97 (33.4)	193 (65.6)	1.00	1.00
	3	90 (31.8)	193 (68.2)	2.37(1.01–5.55)	1.04(0.71,1.53)
	4	60(33.7)	118(66.3)	2.20 (0.94–5.16)	1.06(0.69,1.62)
	5	7 (17.5)	33 (82.5)	2.40(1.00-5.74)	0.48(0.20,1.20)

^*Statistically significant (p<0.05), ** Moderately statically significant (p ≤0.01), *** Highly statistically significant (p≤0.001),^

^COR−Crude odds ratio, AOR−Adjusted odds ratio, CI−confidence interval, 1.00 –Reference^

## Discussion

This study aimed to determine the prevalence of Gastroesophageal reflux disease symptoms, and its associated factors among university students in the Amhara national regional state, Ethiopia. Accordingly, the prevalence of Gastroesophageal reflux disease symptoms in this study was 32.1% (95% CI = 28.7–35.5%). This study finding is consistent with studies from Nigeria (32.8%) [12], Saudi Arabia (33.18%) [25], and India (30%) [9]. This study’s findings were lower than those of previous studies in Sri Lanka (52%) [7], and Saudi Arabia ( 52.6%) [8]. It is, however, higher than studies from Iran (19.3%), [34] and Saudi Arabia (25.9%) [42], Syria (16%) [36], India (14.4%) [10], Italy (26.2%) [11], Brazil (11.8%) [6], and Nigeria (26.34%) [13]. Lifestyle, socioeconomic, and demographic differences in the study population may have contributed to these disparities [4, 54–56].

Regarding associated factors, age, sex, residence, use of antipain, and consumption of soft drinks were significantly associated with GERD symptoms among university students. According to the finding of this study, the participants aged 20–25 years were more likely to have GERD symptoms than those under the age of 20 years old. This finding was consistent with prior findings in the literature [19, 26, 31]. Justification might be that students are under academic pressure, and lifestyle changes may have an impact on the student’s physical and mental health by causing psychological stress and it causes raising gastric acid secretion, decreasing gastric emptying, and enhancing the gastric mucosa’s sensitivity to acid, psychological stress can exacerbate the symptoms of GERD [17].

And also, students are more likely to participate in risky habits like chewing chat, cigarettes smoking, drinking alcohol, sleeping too little, and drinking soft drinks and coffee to relieve stress. Due to decreased lower esophageal sphincter tone, increased acid secretion, irritation of the esophageal mucosa, decreased gastroesophageal motility, and decreased production of bicarbonate-rich saliva, these activities may exacerbate the symptoms of GERD [57–60].

Another factor significantly associated with GERD symptoms is the sex of the respondents. Females had an increased chance of having GERD symptoms compared to males. This finding is consistent with other studies conducted elsewhere [7, 11, 24, 26–30]. The possible reason might be gender-related variation, which influences eating habits and lifestyle factors, as well as hormonal effects (such as progesterone) [54, 61]. Another possible explanation might be females are more prone to stressful conditions than males. Stress activates the hypothalamic-pituitary-adrenal axis to produce cortisol and decreases the production of prostaglandins, increasing stomach acid production, and slowing gastric emptying [62].

Findings from this study revealed that respondents who came from urban were less likely to have GERD symptoms than rural dwellers. One possible reason is that urban dwellers are more aware of good living styles, dietary habits, and easier access to healthcare systems and health-related information than rural dwellers, all of which may reduce the risk of GERD symptoms.

The respondents who used analgesics were more likely to have GERD symptoms compared to those who did not. The finding of this study was consistent with many studies conducted elsewhere [20, 21, 28, 32, 35, 45, 47–50]. The possible reason might be analgesics, directly and indirectly, disrupts physiologic mucosal protection mechanisms in the digestive tract by inhibiting cyclooxygenase enzymes. Besides, analgesics might reduce lower esophageal sphincter pressure, delay emptying of the stomach, and increase acid secretion [4, 50, 63, 64].

Respondents who use soft drinks were more likely to have GERD symptoms than their counterparts. The finding of this study was in line with other findings [8, 9, 18, 24, 25, 38, 39, 41–43]. The possible reason might be soft drinks contain caffeine, gaseous, carbohydrates (sweeteners), and acid [57], which can affect the upper digestive system by causing gastric distention due to the carbonation process, which increases acid reflux by decreasing lower esophageal sphincter pressure, increasing the frequency of transient lower esophageal sphincter relaxation then push stomach acid contents back to the esophagus, increases postprandial acid exposure of the esophagus. Moreover, it might be stimulated by extra gastric acid secretion and acid pocket formation increases gastric acid contents because soft drinks have taken during a meal and after the meal, which results in GERD symptoms [57, 65–67].

This study has limitations. Because the study was cross-sectional, it is difficult to demonstrate cause-and-effect relationships between independent and dependent variables. Because of the significant overlap between dyspepsia and GERD symptoms, dyspepsia cannot be ruled out. Furthermore, because only GERDQ was used for diagnosis, there are a significant number of false positives and false negatives.

## Conclusion

Approximately one-third of university students suffer from gastroesophageal reflux disease symptoms. Age, gender, residence, antipain use, and soft drink consumption were all significantly associated factors. To reduce disease burden, it is recommended to reduce modifiable risk factors such as antipain medication use and soft drink consumption.

## Data Availability

The datasets used and/or analyzed during the current study are available from the corresponding author upon reasonable request.

## Abbreviations

GERD: Gastroesophageal Reflux Disease
GERDQ: Gastroesophageal reflux Disease Questionnaire
